# Cancer-associated fibroblasts: a versatile mediator in tumor progression, metastasis, and targeted therapy

**DOI:** 10.1007/s10555-024-10186-7

**Published:** 2024-04-11

**Authors:** Tianchen Guo, Junfen Xu

**Affiliations:** 1 Women’s Reproductive Health Laboratory of Zhejiang Province, Women’s Hospital, Zhejiang University School of Medicine, Hangzhou, 310006 Zhejiang China; 2 Department of Gynecologic Oncology, Women’s Hospital, Zhejiang University School of Medicine, Hangzhou, 310006 Zhejiang China

**Keywords:** Tumor microenvironment, TGF-β, EMT, Molecular marker

## Abstract

Tumor microenvironment (TME) has been demonstrated to play a significant role in tumor initiation, progression, and metastasis. Cancer-associated fibroblasts (CAFs) are the major component of TME and exhibit heterogeneous properties in their communication with tumor cells. This heterogeneity of CAFs can be attributed to various origins, including quiescent fibroblasts, mesenchymal stem cells (MSCs), adipocytes, pericytes, endothelial cells, and mesothelial cells. Moreover, single-cell RNA sequencing has identified diverse phenotypes of CAFs, with myofibroblastic CAFs (myCAFs) and inflammatory CAFs (iCAFs) being the most acknowledged, alongside newly discovered subtypes like antigen-presenting CAFs (apCAFs). Due to these heterogeneities, CAFs exert multiple functions in tumorigenesis, cancer stemness, angiogenesis, immunosuppression, metabolism, and metastasis. As a result, targeted therapies aimed at the TME, particularly focusing on CAFs, are rapidly developing, fueling the promising future of advanced tumor-targeted therapy.

## Introduction

The tumor microenvironment is a complex ecosystem consisting of a heterogeneous population of cells, including tumor cells and recruited stromal cells [1]. These stromal cells, along with tumor cells, form a solid “castle” both physically and chemically. This abnormal and stiffened structure of tumor vasculature and lymphatic vessels results in interstitial hypoxia, acidic interstitial pH, and drug retention, creating an immunosuppressive and anti-drug barrier in the TME [2]. Furthermore, the heterogenous composition of the TME leads to the release of high levels of chemokines and cytokines, such as transforming growth factor β (TGF-β), interleukin 6 (IL-6), and tumor necrosis factor-α (TNFα). These molecules promote chronic inflammation and extracellular matrix (ECM) remodeling [3].

Cancer-associated fibroblasts (CAFs) are a significant component of the TME. However, their characterization remains imprecise due to their heterogeneity in origin, phenotype, and function. CAFs have been reported to derive from various sources, including quiescent fibroblasts, mesenchymal stem cells (MSCs), epithelial cells, adipocytes, and pericytes, through different but interconnected signaling pathways [4]. Given the diversity of origins and the specific TME of different tumors, CAFs can exhibit multiple phenotypes. In 2018, Bartoschek et al. employed single-cell RNA sequencing to identify several distinct subclasses of breast CAFs, which they classified as vascular CAFs (vCAFs), matrix CAFs (mCAFs), developmental CAFs (dCAFs), and circulating CAFs (cCAFs). These subclasses originate from perivascular cells, resident fibroblasts, malignant cells that have undergone epithelial-to-mesenchymal transition (EMT), and proliferating vCAFs, respectively [5]. As detection technology evolves and interest in CAF research grows, two major CAF subtypes have been widely recognized: myofibroblastic CAFs (myCAFs) involved in ECM remodeling, and inflammatory CAFs (iCAFs) regulating tumor immunity [6]. Several markers have been identified for the identification of CAFs, such as α-SMA, FAP, and COL1A1, but none of these markers is highly specific [7]. Therefore, it is urgently needed to develop a well-established system for accurately distinguishing the heterogenous CAFs.

Tumor-targeted therapy, which has been under development since the early 2000s, has emerged as a viable and remarkable option for cancer patients. These therapies include various approaches such as drugs, viruses and gene therapy [8, 9]. Among the significant components in solid tumors, CAFs play an indispensable role. Consequently, targeted therapies aimed at CAFs have been in prosperous development, ranging from direct CAF depletion to molecular CAF reprograming. However, the majority of these CAF-targeted therapies have faced challenges during clinical trials, likely due to the lack of a specific CAF marker or the occurrence of severe adverse effect.

In this review, we provide a comprehensive definition of CAFs, taking into consideration their peculiar heterogeneity in origin, phenotype, and markers. Furthermore, we make an effort to examine and discuss the diverse regulatory functions of CAFs in tumorigenesis, progression, and metastasis, with a particular focus on the unique molecular pathways involved. Additionally, we endeavor to outline the latest advancements in CAF-targeted therapies, with the ultimate goal of establishing an efficient TME-targeted therapy to overcome the challenges faced in cancer treatment.

## CAF and its heterogeneity

**Fig. 1:**
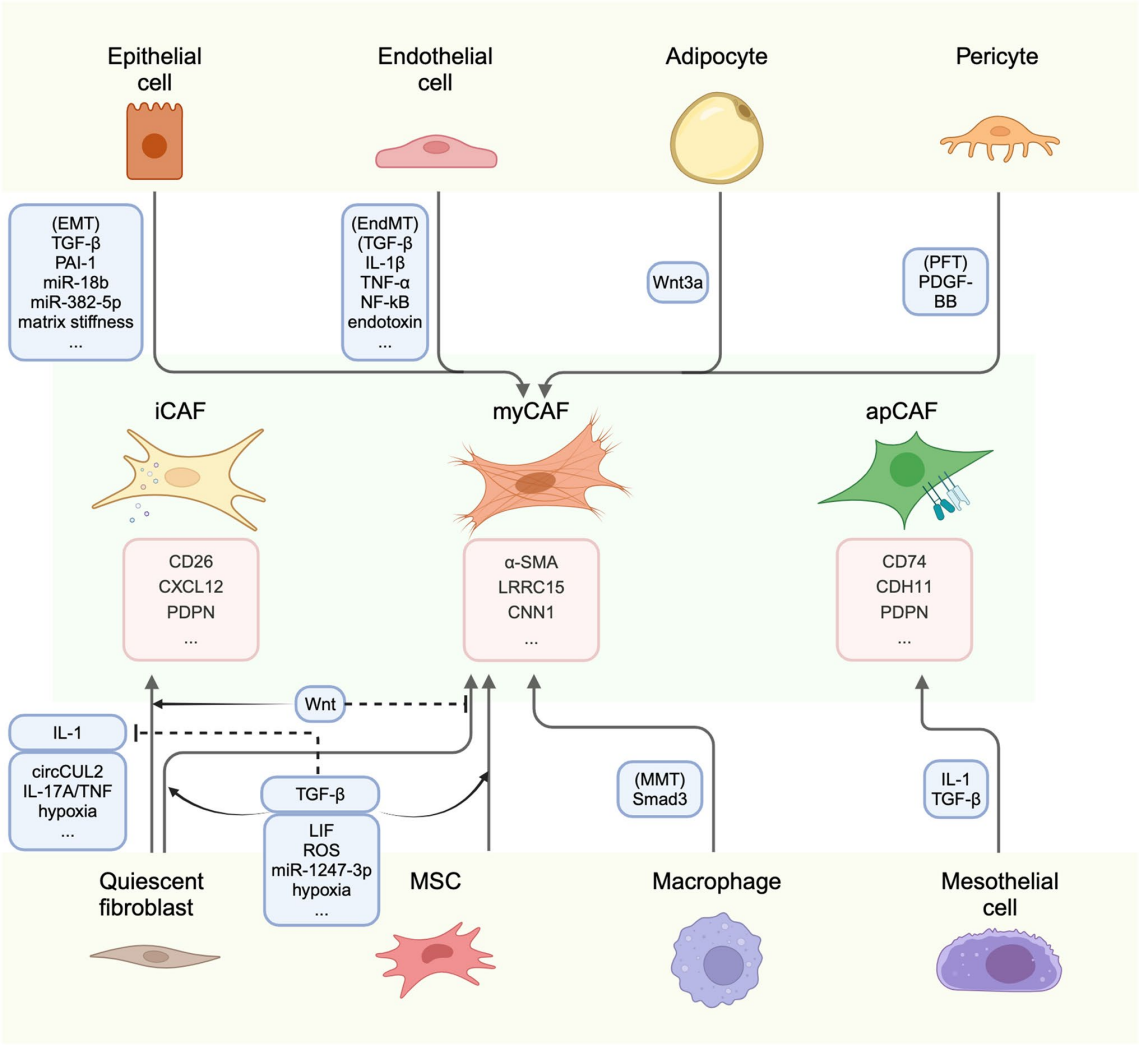
Heterogeneity of CAFs in origin and phenotype. CAFs can be derived from various origins, including quiescent fibroblasts, MSCs, epithelial cells, endothelial cells, adipocytes, pericytes, macrophages, and mesothelial cells, stimulated by respective factors. Moreover, CAFs also exhibit diverse phenotypes, such as myofibroblastic CAFs, inflammatory CAFs, and antigen-presenting CAFs. These distinct phenotypes share several collective markers while also express particular function-associated markers. MSC, mesenchymal stem cell; IL-1, interleukin 1; IL-17A, interleukin 17A; TNF, tumor necrosis factor; TGF-β, transforming growth factor β; LIF, leukemia inhibitory factor; ROS, reactive oxygen species; EMT, epithelial-to-mesenchymal transition; PAI-1, plasminogen activator inhibitor 1; EndMT, endothelial-mesenchymal transition; NF-κB, nuclear factor kappa-B; PFT, pericyte-fibroblast transition; MMT, macrophage-myofibroblast transition; iCAF, inflammatory CAF; myCAF, myofibroblastic CAF; apCAF, antigen-presenting CAF; CXCL12, C-X-C motif chemokine ligand 12; PDPN, podoplanin; α-SMA, α-smooth muscle actin; LRRC15, leucine-rich repeat-containing protein 15; TnC, tenascin-C; CNN1, calponin 1; ANTXR1, anthrax toxin receptor 1; ITGA11, integrin α11; SPARC, secreted protein acidic and rich in cysteine; CDH11, cadherin-11

Fibroblasts are universal and fundamental cells that play a crucial role in building connective tissue. They are spindle-shaped cells with ability of adhesion, signifying their diverse functions in synthesis, construction, and wound healing [10]. In response to tissue injury, quiescent fibroblasts undergo reversible activation to facilitate tissue repair and regeneration. During this process, the activated fibroblasts express α-SMA and vimentin, undergo a transformation into a stellate shape, and exhibit enhanced secretory and migratory function [11]. However, in the context of tumorigenesis, various stimuli drive the injury-activated fibroblasts towards a state of increased secretion and proliferation but reduced contractility. This state is known as CAF [12]. Consequently, CAFs play a role in promoting cancer progression. Unlike the highly preserved resident fibroblasts, CAFs are composed of heterogenous subsets with specific division of duties (Fig. 1).

### The heterogeneity of CAFs in origin

Accumulating evidences have shown that CAFs derive from a heterogenous population of cells. Quiescent tissue fibroblasts and MSCs can be induced to transform into stellate-shaped myofibroblasts when activated by several cytokines, chemokines, and exosomes. For example, TGF-β has been confirmed to activate fibroblasts through both SMAD and non-canonical signaling pathways [13]. Leukemia inhibitory factor (LIF), a member of the IL-6 pro-inflammatory cytokine family, promotes and sustains the pro-invasive conversion of fibroblasts through crosstalk between the JAK1/STAT3 and RhoA/ROCK/MLC2 signaling pathways [14]. Li et al. demonstrated that aggressive cancer cells from both lung cancer and melanoma can produce methylmalonic acid, an oncometabolite increased with aging, which activates fibroblasts through reactive oxygen species (ROS)-activated nuclear factor kappa-B (NF-κB) and TGF-β signaling [15]. Furthermore, Fang et al. reported that hepatocellular carcinoma cells-derived exosomal miR-1247-3p target β-1,4-galactosyltransferases III (B4GALT3), a protein involved in glycosylation, leading to the activation of integrin β1/NF-κB signaling in fibroblasts in lung metastatic niche [16]. In addition to soluble factors, hypoxia has been reported to activate hypoxia-inducible factor 1 (HIF-1) and induce autocrine TGF-β signaling to promote prostate cancer-associated myofibroblast activation [17]. Apart from quiescent fibroblasts and MSCs, there are various non-fibroblastic sources that have the potential to be induced to transdifferentiate into CAFs.

EMT is a pivotal process present universally in both physiological and pathological scenarios. During EMT, a polarized epithelial cell undergoes multiple biochemical changes, leading to the loss of epithelial phenotype and the acquisition of mesenchymal phenotype. The mesenchymal phenotype exhibits enhanced migratory and invading capacity, elevated resistance to apoptosis, and increased productivity of ECM components [18]. EMT is classified into three subtypes. Type-1 EMT is involved in embryo implantation and development, causing neither fibrosis nor an invasive fibroblastic phenotype. Type-2 EMT is associated with wound healing, tissue regeneration, and organ fibrosis in response to stimuli like trauma or inflammatory damage, and the transformed fibroblasts are typically marked by the expression of fibroblast-specific protein 1 (FSP1, also known as S100A4), α-SMA, and collagen I [19]. On the other hand, type-3 EMT usually occurs in epithelial neoplastic cells, playing a critical role in the acquisition of malignant phenotypes with invasion and disseminating capacities [20]. EMT can be initiated and promoted by multiple cytokines, microRNAs, exosomes, and hypoxia. These factors activate various transcriptional regulators, such as Snail, Slug, Twist, and ZEB1/ZEB2, leading to the downregulation of epithelial markers (e.g., E-cadherin, occludin, claudin-1, β-catenin) and the acquisition of mesenchymal markers (e.g., N-cadherin, vimentin, fibronectin) through signaling cascades, including Wnt/β‐catenin, Notch, Sonic hedgehog, NF-κB, receptor tyrosine kinases, PI3K/AKT/mTOR, Hippo, and TGF-β pathways, among others [21]. While it is widely accepted that cancer induces type-2 EMT for “wound” healing, it remains uncertain whether type-3 EMT is involved in the origination of CAFs from resident epithelial cells or if it modulates crosstalk with type-2 EMT through genomic alterations.

Endothelial cells also serve as major sources of CAFs through a process known as endothelial-mesenchymal transition (EndMT) [22]. EndMT is initiated by specific inductors secreted by cancer cells, such as TGF-β, IL-1β, tumor necrosis factor-α (TNF-α), NF-kB transcription factor, and endotoxin [23–25]. TGF-β, among the most common EndMT inducers, exists in three isoforms (TGF-β1, TGF-β2, and TGF-β3). EndMT is primarily induced by the first two isoforms, which bind to the corresponding TGF-β transmembrane receptor type II (TGFβR2) and phosphorylate TGF-β receptor type I (TGFβR1 or ALK5). This activation leads to the phosphorylation of Smad2/3, which forms a complex with Smad4, translocates to the nucleus, and triggers the expression of multiple genes specific for EndMT, including NOTCH1, TWIST1, SLUG, and SNAI1/2. EndMT can also be induced through alternative signaling pathways, such as TGFβ/ALK5/PI3K/Akt pathway.

Adipocytes in TME are also recognized as a source of CAFs, known as cancer-associated adipocytes (CAAs). CAAs undergo dedifferentiation and transition into fibroblast-like cells termed adipocyte-derived fibroblasts (ADFs), with an increased expression of CAF markers such as fibroblast-specific protein-1 (FSP1), but not α-SMA [26]. This process is often initiated by Wnt3a secreted by tumor cells through the Wnt/β-catenin pathway, as discovered in breast cancer by Bochet et al. [27]. Consistent with Bochet’s finding, Iyoshi et al. identified omental adipocyte-derived fibroblast dissected from the metastatic lesions of ovarian cancer and found it exhibiting both mesenchymal stem cell and myofibroblast-like features, according to the expression of CD73 and α-SMA. This pro-tumoral phenotype of omental adipocyte-derived fibroblast is also found to be induced by Wnt3a instead of Wnt5a [28]. The incompatible expression of α-SMA in ADFs may indicate different subpopulations of CAFs originating from adipocytes in different tumor context. Hence, deciphering the heterogeneity of adipocytes-derived CAFs might be instructive for the therapy of fat-rich cancers.

Pericyte, known for promoting angiogenesis and vessel maturation in cancers, can also undergo a transition into CAF-like phenotypes, termed pericyte-fibroblast transition (PFT) [29]. PFT is mainly induced by platelet-derived growth factor type BB (PDGF-BB) and the relevant PDGF-BB-PDGFRβ axis. In the acute phase of benign inflammation, microvascular endothelial cells contribute to most circulating PDGF-BB, while during the chronic inflammation of cancer, the tumor bulk can produce a high level of PDGF-BB, promoting angiogenesis and initiating PFT [30]. Hosaka et al. induced mouse pericytes with PDGF-BB, and whereafter found it losing Ng2 expression while gaining of Fsp1 and α-Sma in pericytes, additionally discovering the function of stimulating tumor growth and metastasis in PDGF-BB-primed pericytes [31].

Furthermore, there are several other origins of CAFs, such as a subset derived from the macrophage lineage found in non-small-cell lung carcinoma, regulated by Smad3-induced macrophage-myofibroblast transition (MMT) [32]. Mesothelial cells can also transform into a typical antigen-presenting CAFs (apCAFs) when induced by IL-1 and TGF-β, discovered in pancreatic cancer [33]. The exact origin of CAFs still lacks precise elaboration, and further approaches like lineage tracing, fate-mapping, pseudotime analysis, and RNA velocity may help uncover new primogenitors of CAFs.

### The heterogeneity of CAFs in phenotype

Previous studies have indicated that α-SMA, FAP, vimentin, FSP1 (also known as S100A4), PDGFRα, and PDGFRβ could serve as markers to define CAFs. These studies have also shown that CAFs play a tumor-promoting function in various ways. However, the lack of a specific CAF marker and the discovery of tumor-restraining CAFs have driven research on the functional phenotypes of CAFs [11, 34]. Due to the various origins of CAFs, it is evident that CAF subpopulations exhibit significant diversity. The proximity of CAFs to cancer cells and the presence of different soluble factors in the TME may also contribute to this heterogeneity [35]. With the advancement of single-cell RNA sequencing, researchers have identified multiple and distinct populations of CAFs with characteristic markers. Table 1 shows several markers found in CAFs and its subpopulations; however, none of these markers have been strictly proven to be specific.

**Table 1:** List of CAF markers with the functional features and expression in specific subtypes

Marker	Name	Features	Expression in CAF subpopulations	Ref
FSP1	Fibroblast-specific protein-1, S100A4	A reliable marker to detect quiescent, non-proliferating (Ki67^−^) fibroblasts; non-FAP expressing CAF subpopulations observed	Normal fibroblasts	[52]
PDGFR	Platelet-derived growth factor receptor	Universal in fibroblasts, associated with tamoxifen resistance	Normal fibroblasts	[53]
VIM	Vimentin	Associated with EMT	Normal fibroblasts	[54]
FAPα	Fibroblast activation protein α	The most viable CAF markers for potential clinical application	Nonspecific CAF	[52, 53, 55]
Cav-1	Caveolin-1	Loss of caveolin-1 Induces CAF phenotype	Nonspecific CAF	[56]
COL1A1	Collagen type I α1	COL1A1/integrin β1 promotes tumor migration and invasion	Nonspecific CAF	[57]
BIRC5	Survivin	Regulate the interplay between mitosis, apoptosis, and autophagy cancer cells	Nonspecific CAF	[58]
TWIST1	Twist-related protein 1	An EMT inducer and a suppressor of CAFs’ senescence	Nonspecific CAF	[59]
GPR77	G protein-coupled receptor 77	Correlated with chemoresistance via cancer stemness	Nonspecific CAF	[60]
NG2	Neural-glial antigen 2	Also expressed in pericytes	Nonspecific CAF	[61]
CD90	Thy-1	Associated with cell–cell and cell–matrix interactions;	Nonspecific CAF	[62]
α-SMA	α-Smooth muscle actin, smooth muscle aortic alpha-actin (ACTA2)	Associated with wound healing, causing myofibroblast contractility	myCAF	[5]
LRRC15	Leucine-rich repeat-containing protein 15	A highly restricted marker associated with anti-tumor T cell immunity	myCAF	[63]
TnC	Tenascin-C	A member of ECM; promote tumor migration and invasion	myCAF	[64]
CNN1	Calponin 1	Upregulated during myofibroblastic differentiation	myCAF	[65]
ANTXR1	Tumor endothelial marker 8 (TEM8)	A highly conserved transmembrane receptor expressed on CAFs, endothelium, and pericytes	myCAF	[66]
ITGA11	Integrin α11	Receptor for collagen remodeling and CAF migration; expressed in a subset of non-pericyte-derived CAFs	myCAF	[67]
SPARC	Secreted protein acidic and rich in cysteine	Downregulation of SPARC promotes EMT	myCAF	[68, 69]
RGS5	Regulator of G-protein signaling 5	A surface marker of pericytes; associated with angiogenesis and metastasis	myCAF-like in bladder carcinoma, iCAF in cholangiocarcinoma	[61, 70, 71]
CD26	Dpp4	CD26^+^ NFs transit into pro-tumorigenic iCAFs	iCAF	[72]
CXCL12	C-X-C motif chemokine ligand 12	Interacting with T cells via CXCL12-CXCR4/CXCR7	iCAF	[73]
PDPN	Podoplanin	Associated with immune cell infiltration and recruitment of dendritic cells	iCAF, apCAF	[33, 74]
CD74	MHC class II invariant chain, Ii	Associated with presenting antigens to CD4^+^ T cells and the modulation of immune response	apCAF	[47, 75]
CDH11	Cadherin-11	Promote immunosuppression and ECM deposition	apCAF	[76]

myCAFs are a subpopulation of CAFs that exhibit similarities with myofibroblasts involved in the wound healing process. Due to these similarities, myCAFs have been reported to play a major role in producing ECM components and remodeling ECM [36]. Based on the heterogenous expression of SDC1, LAMP5, and CD9, Kieffer et al. further divided ANTXR1^+^ myCAFs into different subtypes: SDC1^+^LAMP5^−^ ecm-myCAF, which expresses genes coding for ECM proteins; LAMP5^+^SDC1^+/−^ TGFβ-myCAF, expressing the TGFβ pathway; SDC1^−^LAMP5^−^CD9^+^ wound-myCAF, expressing wound healing proteins; and acto-myCAF, programming acto-myosin pathway. This highlights the high heterogeneity in the myCAF phenotype [37]. However, it has been shown that the phenotype of myCAF is flexible rather than fixed. Mosa et al. demonstrated that high and low levels of Wnt induce myCAF and iCAFs subtype, respectively, indicating the regulation of tumor growth and malignancy through CAF subtypes transition under the influence of the Wnt/β-catenin pathway [38].

iCAFs have been identified in multiple cancers, indicating their universal presence within CAF clusters. It has been demonstrated that iCAFs share similar transcriptional profiles and signaling pathway activation with senescent fibroblasts [34]. In pancreatic cancer, iCAFs derived from pancreatic stellate cells (PSCs) exhibit significantly lower α-SMA expression but intensely high expression of inflammatory cytokines, such as IL-6 and IL-11, compared to the homologous myCAFs [39]. Similarly, in bladder carcinoma, single-cell sequencing in COL1A1^+^ fibroblasts has revealed a PDGFRA^+^ iCAF cluster that exhibit strong expression of cytokines and chemokines, including CXCL12, IL6, CXCL14, CXCL1, and CXCL2, compared to RSG5^+^ myCAF cluster. The pro-proliferation effect of this cluster has been confirmed [40]. On the other hand, in cholangiocarcinoma, iCAFs identified in COL1A1^+^ fibroblasts show high expression of RSG5, Lrat, and Reln, with enrichment of inflammatory, growth factor, and antigen-presentation genes as well as receptor-ligand, growth factor, and cytokine activity pathways [41]. These findings collectively confirm the inflammatory cytokine-secreting property of iCAFs while also indicating the heterogeneity of iCAFs in different organs.

The generation of the inflammatory phenotype in iCAFs has been demonstrated to be induced by various factors, including cytokines, circRNAs, T cells, and hypoxia. Biffi et al. illustrated that IL-1 induces leukemia inhibitory factor (LIF) expression to activate JAK/STAT signaling pathways, generating an iCAF phenotype, and TGF-β antagonizes this process by downregulating IL1R1 expression, promoting differentiation into myCAFs [42]. Wnt activity in CAFs is also associated with distinct subtypes, where low and high levels induce an iCAF subtype or contractile myCAFs, respectively [38]. Besides cytokines, Zheng et al. found that the upregulation of circCUL2 expression in normal fibroblasts (NFs) induces the iCAF phenotype and promotes tumorigenesis and metastasis of pancreatic ductal adenocarcinoma (PDAC) cells via miR-203a-3p/MyD88/NF-κB/IL6 axis [43]. Tc17, a novel protumorigenic CD8^+^ T cell subtype in PDAC, has been demonstrated to induce iCAF differentiation via synergism of IL-17A and TNF [44]. Furthermore, Schwörer et al. revealed that hypoxia drives fibroblasts to gain an inflammatory gene expression signature and synergizes with cancer cell-derived cytokines to promote an iCAF phenotype in an HIF-1α dependent fashion, and experimental evidence supporting this finding [45, 46].

In 2019, Elyada et al. unveiled a new population of CAFs expressing MHC class II and CD74 but lacking classical co-stimulatory molecules, leading to their designation as antigen-presenting CAFs (apCAFs) [47]. These apCAFs were found to originate from mesothelial cells through mesothelial-mesenchymal transition, which is induced via IL-1/NF-kB and TGF-β/Smad signaling pathway [33, 48]. The presence of MHC II molecules on apCAFs enables them to present antigens to CD4 + T cells. However, due to the absence of co-stimulation molecules (such as CD40, CD80, and CD86) on apCAFs, they promote the formation of regulatory T cells, which may suppress the immune response [49]. Although it has been observed that patients with a high abundance of apCAFs in PDAC have a decreased survival probability, the specific mechanism by which apCAFs promote cancer progression is still not fully understood [50]. Future research and evidence are required to elucidate the exact role and impact of apCAFs in cancer development and progression.

In addition to the well-known subtypes of CAFs, such as myCAF, iCAF, and apCAF, there are other rare subtypes of CAFs that have been identified through single-cell RNA sequencing, highlighting the high diversity of CAF population and the different criteria used for their classification. For example, there are vascular CAFs (vCAFs) characterized by their high expression of angiogenic or microvasculature-associated genes, like CAD146. Another subtype is the PLA2G2A^+^ metabolic CAFs (meCAFs), which have been found to be correlated with the presence of immune cells [51]. As CAF-detecting techniques continue to advance, it becomes increasingly important to establish a uniform classification system for CAFs to facilitate more precise and targeted therapies. The heterogeneity of CAFs underscores the need for a comprehensive understanding of their distinct subtypes and functional roles in the TME, which may ultimately lead to more effective treatment strategies for cancer patients.

## Methodologies in CAF research

Research on CAFs presents challenges due to their high heterogeneity and context-dependent nature. The expanding field of TME research has led to the gradual establishment of a comprehensive methodology for studying CAFs. In *in vitro* investigations, primary CAFs derived from patients are the preferred choice due to their accessibility and similarity to TME *in vivo*. Protocols for establishing CAFs *in vitro* from surgically resected tissues involve steps such as tissue extraction, digestion, CAF dissociation, and incubation [77]. However, limitation persists, including the loss of heterogeneity in isolated CAFs and the limited number of passages for primary CAFs (usually up to 20–25 passages) [78]. To address senescence during cell expansion, some researchers use lentivirus containing human telomerase reverse transcriptase (hTERT) to immortalize patient-derived CAFs [79]. In addition to *in vitro* studies, CAF-targeted animal models have been developed. For instance, transgenic α-SMA-tk mice are created by ligating a fragment with α-SMA (or other CAF-specific genes) promoter and a truncated version of the herpes simplex 1 virus thymidine kinase (HSV1-tk). Subsequent injection of ganciclovir induces selective depletion of α-SMA myofibroblasts *in vivo* [80]. Beyond genetic engineering, direct tumor injection of pharmaceuticals and orthotopic xenograft implantation with control/treated CAFs are also effective in studying CAF biology [81].

Detecting the heterogeneity of CAFs is crucial and single-cell analysis has been instrumental in understanding distinct CAF subpopulations and their markers. With the rapid development of single-cell analysis techniques, including multiomic analysis, spatial transcriptomics, and proteomics, more sophisticated methods are applied in CAF detection [82, 83]. Flow cytometry, immunohistochemistry, and immunostaining are also vital for CAF taxonomy. Commonly used probes like α-SMA, vimentin, FAP, FSP1, and PDGFR serve as reliable CAF markers when used in combination [84].

The highlight of CAF research lies in understanding the crosstalk between CAFs and other cell types, particularly cancer cells. Coculture analysis is the most efficient way to explore the interactions. However, traditional 2-dimensional cell line coculture system and patient-derived tumor xenografts model have limitations in mimicking human stromal compartments, immune microenvironment, and organ-specific functions for detailed research [85, 86]. The invention of 3-dimensional organoid technology allows *in vitro* tumor research to closely replicate the structural and functional aspects of counterpart organs, facilitating personalized precision oncology [87]. Despite the challenges, advanced models like organoid-on-a-chip have demonstrated comparable responses to therapies with greater precision in predicting outcomes in CAF research [88]. As tumor research technologies evolve, it is expected that more accurate and efficient techniques for CAF research will emerge.

## CAF in cancer progression and metastasis

### CAFs contribute to cancer stemness

Cancer stemness refers to the self-renewal and propagation abilities of cancer stem cells (CSCs), which play a critical role in tumor aggressiveness, drug resistance, and metastasis [89]. CSCs can be identified by several markers, including CD44, CD24, CD133, LGR5, SOX2, AQP5, ESA, PAF1, and CXCR4, although none of these markers is highly specific [90–94]. As a central component of the TME, CAFs are believed to interact with CSCs and maintain a favorable tumor niche, mainly through paracrine signaling [95]. A group of CAF-derived molecules has been found to promote cancer stemness. For instance, Su et al. identified a CD10^+^GPR77^+^ subset of CAFs in breast and lung cancer, driven by NF-kB activation, which induces CSC enrichment by secreting IL-6 and IL-8 [60]. Ma et al. found that interferon secreted from bladder cancer cells can induce SLC14A1^+^ CAFs, which in turn promote stemness of bladder cancer cells via WNT5a/β-catenin pathway [96]. In hepatocellular carcinoma, CAF-derived hepatocyte growth factor (HGF) enhances cancer cell stemness through the extracellular signal-regulated kinase (ERK)1/2-FRA1-HEY1 signaling pathway [95]. Additionally, CAF-secreted exosomes have been shown to sustain cell stemness in various cancers [97, 98]. Furthermore, CAFs indirectly recruit myeloid-derived suppressor cells (MDSCs) and enhance the stemness of CD33^+^ MDSCs in a FAP-dependent paracrine manner [99]. Thus, targeting these paracrine pathways could be a potentially effective approach to combat tumor stemness. The WNT signaling pathway represents a promising target for specific therapy. Canonical WNT signaling supports the rapidly cycling CSCs, while noncanonical WNT signaling supports the quiescent CSCs [100]. Moreover, WNT signaling broadly mediates the communication between CAFs and CSCs, further emphasizing its importance in tumor stemness regulation.

### CAFs promote angiogenesis

Angiogenesis is a critical process whereby tumors develop new blood vessels to obtain an increased supply of oxygen and nutrients [101]. Hypoxia has been identified as a key driver for tumor angiogenesis. Under hypoxic conditions, cancer cells secrete vascular endothelial growth factor A (VEGFA) which binds to VEGF receptor 2 (VEGFR2) on nearby endothelial cells (ECs) of blood vessels or circulating bone marrow-derived endothelial progenitor cells, and triggering angiogenesis [102]. This process involves the breakdown of the basal lamina and ECM, proliferation of ECs, growth of new vascular sprouts, and vessel maturation. Other signaling molecules, such as delta ligand-like 4 (DLL4) and angiopoietin 2 (ANGPT2), also play crucial roles in angiogenesis [103]. CAFs, originating from the chronic wound-healing response within the tumor, secret pro-angiogenic growth factors that promote angiogenesis. These factors include VEGFA, CXC-chemokine ligand 12 (CXCL12), fibroblast growth factor 2 (FGF2), and platelet-derived growth factor (PDGF) [104]. CXCL12, also known as stromal cell-derived factor 1 (SDF-1), has been shown to enhance tumor growth and angiogenesis through the CXCL12/CXCR4 pathway [105]. This binding initiates divergent signaling pathways, including G-protein/PI3K/AKT/NF-κB axis and Ras-MEK1/2-Erk1/2 axis, resulting in various angiogenic responses [106]. Similarly, FGF2, a member of the heparin-binding growth factor family, binds to FGF receptors (FGFRs) and triggers multiple pro-angiogenic activity, while also participating in crosstalk with VEGF [107]. Additionally, the PDGF/PDGF receptor (PDGFR) signaling plays a significant role in connective tissue development and wound healing [108]. Studies have demonstrated that CAFs with upregulated PDGF-C induce angiogenesis even when VEGF is inhibited, suggesting that the PDGF/PDGFR pathway might compensate for the inhibition of VEGF-mediated angiogenesis [109].

In addition to the direct activation of paracrine ways, CAFs have been reported to promote angiogenesis through various indirect mechanisms. One crucial biomechanical characteristic of the TME, driven by stromal cells, particularly CAFs, is matrix stiffness [110]. CAFs secrete lysyl oxidase (LOX), an enzyme that catalyzes the covalent cross-linking of collagens and elastin, contributing to the determination of matrix stiffness [111]. Additionally, CAFs secrete lysyl hydroxylase 2 (LH2), which induces hydroxylysine aldehyde-derived collagen cross-links in the ECM, further increasing matrix stiffness [112]. Numerous studies have demonstrated a link between matrix stiffness and the production of VEGF. For instance, Sack et al. found that on harder ECM surfaces, endothelial cells exhibit an increased capability of binding VEGF and reduced VEGF internalization, regulated by integrins β1 [113]. Li et al. unraveled a matrix stiffness/integrins β1/Piezo1 activation/Ca^2+^ influx/HIF-1α ubiquitination/VEGF pathway in hepatocellular carcinoma angiogenesis, with involvement of CXCL16 and IGFBP2 pathways [114]. However, contradictory results were reported by Bao et al., who discovered a YAP/RUNX2/SRSF1 axis in neuroblastoma angiogenesis, wherein VEGF_165_ secretion is repressed with increasing matrix stiffness [115]. Notably, the disparity in results may be attributed to variations in the stiffness levels of the gels used in the research of Bao et al. (1 kPa, 8 kPa, and 30 kPa, respectively), which might as well be scaled up to a minished range so as to simulate an actual intratumoral microenvironment. Hence, more precise experiments are needed to clarify the functional impact of matrix stiffness on angiogenesis.

As mentioned earlier, hypoxia is a key driver of tumor angiogenesis. Hypoxia-inducible factor (HIF) transcription factors are pivotal in hypoxia signaling in cancer and stromal cells. They translocate to the nucleus in response to the absence of oxygen and activate the expression of hypoxia-related genes, including VEGF [116]. CAFs activated by hypoxic TME can induce abnormalities in the blood vessel by secreting various proangiogenic factors [117]. Furthermore, CAFs produce soluble factors like CCL5, triggering the HIF-1α pathways to promote angiogenesis [118].

### CAFs mediate immunosuppression

Chronic inflammation, immune cell infiltration, and evasion of cancer cells from the immune response are considered some of the hallmarks in cancer progression [35]. Previous studies have established the paradoxical role of the immune system in both promoting and restraining cancer, referred to as “cancer immunoediting.” This dynamic process consists of three sequential phases: elimination, equilibrium, and escape [119]. During the elimination phase, the innate and adaptive immune systems cooperate to recognize and eradicate dysplastic cells before they can develop into clinically detectable tumors. However, if a few variant cancer cells acquire poorly immunogenic or immunoevasive properties that enable them to survive the immune attack, they may enter the equilibrium phase. In this phase, neoplastic cells are still restricted, and their cellular immunogenicity is shaped by the adaptive immune system, primarily involving T cells and related cytokines. As the edited cancer cells face constant immune selection pressure during the equilibrium phase, they may develop immunosuppressive and/or immunoevasive phenotypes, ultimately leading to immune escape. Once in the immune escape phase, the cancer cells are no longer restricted by the immune system, leading to uncontrolled growth, clinically apparent tumors, and even metastasis [120]. Despite its importance, the intricate mechanism of cancer immunoediting is still not fully understood, which presents a challenge for effective immunotherapy targeting cancer.

CAFs, as the major components in the TME, have been reported to mainly exert an immunosuppressive function in facilitating cancer immune evasion. TGF-β, which can be secreted by CAFs, is a significant mediator in the regulation of the immune microenvironment. TGF-β signaling is known to impact T cell differentiation and proliferation by dampening the stimulation of specific transcription factors triggered by Ca^2+^ influx [121]. In a T cell excluded cohort of ovarian tumor, the upregulation of TGF-β and the activation of stroma are identified as important mechanisms of T cell exclusion. TGF-β can reduce MHC-I expression in ovarian cancer cells *in vitro* and also activate fibroblasts to induce extracellular matrix production, constructing a physical barrier to hinder T cell infiltration [122]. Additionally, TGF-β has been demonstrated to suppress dendritic cells, inhibit the development of cytolytic natural killer cells (NK cells), and reduce their secretion of IFN-γ. Moreover, it polarizes macrophages towards the M2 phenotype with anti-inflammatory, immune-suppressive, and pro-angiogenic functions [123, 124]. Apart from TGF-β secretion, CAF-derived CXCL12 is a powerful chemokine involved in immunosuppressive regulation. It reduces CD8^+^ T cells migration, sequestering them from the panstromal compartment, and inhibits NK cell proliferation, maintaining them in a quiescence state [125, 126]. Another essential molecule secreted by CAFs in immune microenvironment is IL-6, which is abundantly expressed in iCAF subtype [127]. IL-6 is associated with the accumulation of tumor-infiltrating lymphocytes and plays a role in regulating the survival, activation, and function of neutrophils through the IL-6/STAT3/PD-L1 signaling pathway [128, 129]. Moreover, CAFs also secret inhibitory immune checkpoints (iICPs) to create an immunosuppressive milieu in the TME, including PD-1 and LAG3 [130]. These pieces of evidence strongly indicate that CAFs play a crucial role in assisting the tumor’s immune escape process.

### CAFs dedicate in metabolic changes in cancer

Despite living in a nutrition-limited TME, cancer cells are highly skilled in perpetual proliferation, which is supported by the metabolic change that occur in the TME. About a century ago, Warburg et al. observed that even in the presence of abundant oxygen, cancer cells exhibited an enhanced and accelerated conversion of glucose to lactate for ATP formation, a phenomenon known as the “Warburg Effect” [131]. Warburg attributed this phenomenon to the dysfunction of mitochondria in tumor cells. As further research in cancer metabolism progressed, it became evident that, in contrast to the Warburg Effect, some tumor cells retain the ability to utilize mitochondria and undergo oxidative phosphorylation (OXPHOS), indicating the dynamic nature of the Warburg Effect in different TME. In some cases, CAFs are reported to adapt their metabolism in response to factors secreted by cancer cells. In this scenario, CAFs switch to aerobic glycolysis and produce high levels of energy‑rich intermediate metabolites, which are then transferred to cancer cells to fuel the mitochondrial tricarboxylic acid cycle and OXPHOS, leading to the production of ATP for cancer cell proliferation [132]. This phenomenon is referred to as the “Reverse Warburg Effect,” acting as a supplementary mechanism to the classic Warburg Effect.

The Reverse Warburg Effect is strongly driven by cancer cell-promoted oxidative stress. Cancer cells release reactive oxygen species (ROS), which reciprocally elevate oxidative stress in the stromal components, enabling autophagosomes to fuse with lysosomes and leading to the destruction of mitochondria in CAFs. This process also results in the degradation of caveolin-1 (Cav-1) through the HIF-1α/ NF-κB pathway [56, 133]. The downregulation of Cav-1 in CAFs, in turn, elevates ROS levels in cancer cells, creating a positive feedback loop that further enhances oxidative stress and impedes NF-kB pathway [134]. TGF‑β, which has a firm and universal association with cancer metabolism, can also regulate the expression of α‑SMA and NOX4 in fibroblasts, thereby influencing ROS levels and stimulating oxidative stress [135]. Through the Reverse Warburg Effect, oxidative cancer cells can receive lactate from hypoxic cancer cells. In addition, CAFs experience oxidative stress due to the cancer cells-secreted ROS, which triggers aerobic glycolysis. As a result, CAFs produce lactate and pyruvate, which can be utilized for further metabolic process in adjacent oxidative cancer cells. Although the transmission of ROS has been substantiated in the Reverse Warburg Effect, there have been few studies elucidating the mechanism by which cancer cells and CAFs perform initiation and adaption to such metabolic changes. Still, targeting the Reverse Warburg Effect, either in cancer cells or through CAFs, is theoretically feasible and could potentially decrease cancer cell metabolism. By disrupting this metabolic interplay between cancer cells and the stromal microenvironment, new therapeutic strategies may be developed to target cancer metabolism and inhibit tumor growth effectively.

### CAFs facilitate cancer metastasis

**Fig. 2:**
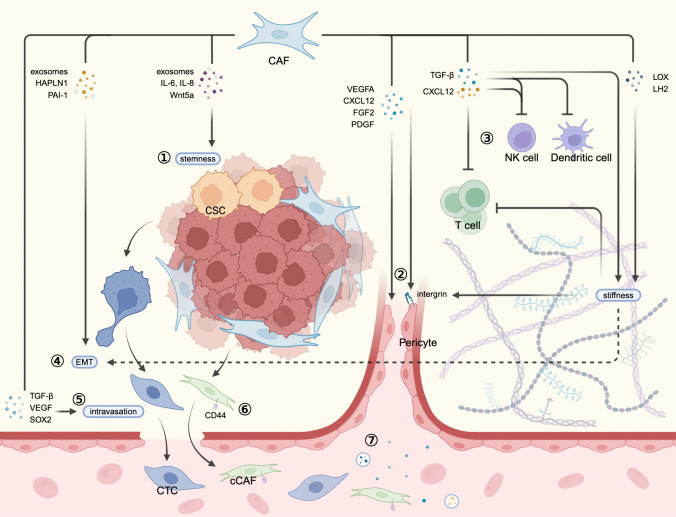
The role of CAFs in cancer progression and metastasis. ① Several specific subsets of CAFs secrete exosomes, ILs, and Wnt5a to promote cancer stemness. ② CAFs directly produce soluble factors, including VEGFA, CXCL12, FGF2, and PDGF, to trigger angiogenesis by binding to the receptors on endothelial cells. On the other hand, CAFs secrete of LOX and LH2 to enhance ECM stiffness, which facilitates VEGF/VEGFR interaction via integrins. ③ CAF-derived TGF-β can impact T cell differentiation and proliferation, dampen MHC-mediated immune identification, and reduce T cell infiltration through fortifying matrix stiffness. Moreover, TGF-β can also suppress dendritic cells and inhibit the development of cytolytic NK cells. CXCL12 secreted from CAFs can reduce T cells migration and inhibits NK cells proliferation. ④ Various factors produced from CAFs can enhance cancer cell migration and invasion by inducing EMT. Additionally, CAF-promoted matrix stiffness contributes to EMT. ⑤ CAFs secret TGF-β, VEGF, and SOX2 to regulate the intravasation of blood vessels. ⑥ A cCAF subtype is involved in the metastasis through the blood vessels, marked by CD44. ⑦ Metastasis-associated cytokines and exosomes derived from CAFs in primary tumor facilitate the formation of the distant PMN. CAF, cancer-associated fibroblast; HAPLN-1, hyaluronan and proteoglycan link protein 1; PAI-1, plasminogen activator inhibitor-1; IL-6, interleukin 6; IL-8, interleukin 8; CSC, cancer stem cell; VEGFA, vascular endothelial growth factor A; CXCL12, CXC-chemokine ligand 12; NK cell, natural killer cell; LOX, lysyl oxidase; LH2, lysyl hydroxylase 2; EMT, epithelial-to-mesenchymal transition; CTC, circulating tumor cell; cCAF, circulating CAF

Cancer metastasis is a complex process involving multiple stages. It begins with tumor cells migrating and invading nearby tissues, followed by intravasation, circulation, and extravasation, and ultimately colonization at the target site [136]. CAFs play a significant role in promoting metastasis through both paracrine signaling ways and physical interactions (Fig. 2).

The motility of cancer cells is closely related to their ability to migrate and invade, which is often facilitated by the process of EMT. EMT is characterized by the loss of polarity, adhesion, and tight junctions, leading to cancer cells adopting a mesenchymal phenotype that promotes migration and invasion. However, it remains controversial whether EMT is essential for every metastatic event [137, 138]. CAFs have been shown to enhance cancer cell migration and invasion by secreting various factors, including chemokines and exosomes. For example, in gastric cancer, CAFs activated by TGF-β1/Smad2/3 signaling can highly express hyaluronan and proteoglycan link protein 1 (HAPLN1), promoting tumor migration and invasion [139]. In esophageal squamous cell carcinoma, plasminogen activator inhibitor-1 (PAI-1) derived from CAF-like cells enhances migration and invasion abilities through the Akt-Erk1/2 signaling pathways via the PAI-1/low-density lipoprotein receptor-related protein 1 (LRP1) axis [140]. CAF-secreted exosomes containing miR-18b and miR-382-5p have also been reported to promote cancer cell migration and invasion through EMT [141, 142]. Additionally, CAFs can promote EMT induction by increasing matrix stiffness signaling mediators such as TWIST1/G3BP2 pathway and EPHA2/LYN/TWIST1 pathway [143, 144]. Apart from inducing EMT, CAFs can directly drive cancer cell migration through physical forces. Labernadie et al. identified a mechanism in which CAFs exert physical force on cancer cells through the heterophilic adhesion involving N-cadherin on the CAF membrane and E-cadherin on the cancer cell membrane, mediated by β-catenin recruitment and α-catenin/vinculin interaction [145]. Erdogan et al. demonstrated that CAFs produce and align a fibronectin (Fn)-rich matrix via the nonmuscle myosin II/PDGFRα/α5β1-integrin/Fn pathway to mediate CAF-cancer cell association and directional migration [146]. Additionally, CAFs express membrane-anchored metalloproteinases (MT1-MMPs) that have collagenolytic effect, facilitating tumor cell penetration of connective tissue barriers and trafficking within the three-dimensional ECM [147].

Intravasation is a crucial process that occurs before tumor cells can enter the circulation and spread to distant sites. During angiogenesis, the formation of new blood vessels, the vessels are often considered immature, lacking proper junctional contacts between endothelial cells, and are leaky and vulnerable due to abnormal pericyte coverage. These features enable cancer cells to easily intravasate through the blood barrier [148]. Several factors, such as TGF-β, VEGF, and SOX2, have been shown to play roles in regulating both the intravasation and extravasation processes during metastasis [149, 150]. CAFs not only promote hematogenous metastasis (metastasis through the blood vessels) but also play a role in facilitating lymphatic metastasis (metastasis through the lymphatic vessels). This promotion of lymphatic metastasis has been reported to involve various signaling pathways, such as periostin/integrin/FAK/Src/VE-cadherin pathway, VEGFC/VEGFR3 pathway, and IL-6/IL-6R pathway [151–153].

Despite their role in motivating tumor cells, CAFs themselves are not quiescent. In 2015, Ao et al. examined a functional subpopulation of CAFs in the peripheral blood of patients with metastatic breast cancer, referred to as circulating CAF (cCAF), the presence of which was associated with clinical metastasis [154]. Sharma et al. also detected this heterotypic cluster of cells in patient blood and preclinical mouse models of breast cancer, and they found that CD44, an adhesion and stemness marker, might be an important mediator in this context [155]. Furthermore, Hurtado et al. utilized a metastasis model in zebrafish and observed that CAFs exert a pro-survival and pro-proliferative effect on circulating tumor cells (CTCs) when they remain joined as cell clusters. This clustering led to production of soluble factors associated with breast cancer cell survival and proliferation [156]. Identifying and targeting cCAFs at an early stage of tumor development could be a potent therapeutic approach to reduce cancer metastasis and relapse, especially since the detection of cCAFs in patients with localized breast cancer has also been reported [154].

CTCs that extravasate at the target site face a challenging microenvironment that is often hostile for their survival. Interestingly, even before the metastasis process begins, the host metastasis site microenvironment at the future metastatic site has already been selectively modified by the remote primary tumor. This modified microenvironment is referred to as pre-metastatic niches (PMN) [157]. The formation of PMN is largely attributed to cytokines and exosomes released by the tumor and the TME. CAFs play a dual role in activating the PMN. On the one hand, metastasis-associated factors derived from CAFs in primary tumor facilitate the formation of pre-metastatic niche. For example, a long non-coding RNA called LncSNHG5 expressed in breast CAFs is found to mediate angiogenesis and vascular permeability in the PMN of the lung through the lncSNHG5-ZNF281-CCL2/CCL5 signaling axis [158]. Similarly, extracellular vesicles (EVs) derived from CAFs in salivary adenoid cystic carcinoma induce remarkable changes in lung fibroblasts, enhancing their tumor-permissive abilities. The uptake of CAF EVs by lung fibroblasts is mediated through integrin α2β1 [159]. On the other hand, fibroblast activation into CAF is recognized as the initial phase during PMN formation. Research by Pein et al. has shown that breast cancer cells secrete IL-1α and IL-1β, which induce lung fibroblasts to produce CXCL9 and CXCL10 via NF-κB signaling, leading to inflammatory phenotypic changes in lung fibroblasts [160]. Besides cytokines, Ji et al. discovered that primary colorectal tumors release integrin beta-like 1 (ITGBL1)-enriched EVs, which stimulate the TNFAIP3-mediated NF-κB signaling pathway to activate remote fibroblasts and transform them into CAFs. These CAFs subsequently induce the formation of the PMN by secreting proinflammatory cytokines such as IL-6 and IL-8 [161]. After activation, CAFs play critical roles in ECM remodeling, metabolic changes, immunosuppression, and angiogenesis, all of which contribute to the formation of the PMN, as discussed earlier [162].

### CAFs reinforce therapeutic resistance

Therapeutic resistance in cancer often leads to a poor prognosis in patients, and the underlying mechanisms behind it remain complex and dynamic. Konieczkowski et al. proposed a convergence-based framework for understanding cancer drug resistance, with pathway reactivation, pathway bypass, and pathway indifference being major causes of resistance [163]. Besides genomic changes in tumor cells, the involvement of CAFs has been extensively demonstrated in cancer therapeutic resistance, with their role being multifaceted.

CAF’s influence on the mechanical TME can promote matrix stiffness, thereby reducing the infiltration of chemical drugs. For instance, gastric CAFs expressing calponin 1 activate ROCK1/MLC pathway, leading to increased matrix stiffness and contributing to 5-fluorouracil (5-Fu) resistance in cancer cells by activating YAP [164]. CAF-derived exosomes also play a significant role in mediating cancer therapy resistance in the TME [165]. Annexin A6 in CAF-derived EVs can activate the integrin β1-focal adhesion kinase (FAK)-YAP signaling pathway, leading to the formation of a tubular network in the ECM, reinforcing chemotherapeutic resistance [166]. In breast cancer, CAF-derived circulating EVs containing the full mitochondrial genome promote estrogen receptor (ER)-independent OXPHOS, inducing therapy-induced dormant cancer stem-like cells and leading to endocrine therapy resistance [167]. Targeting the YAP signaling pathway may hold promise in overcoming the mechanical resistance encountered in targeted therapy. In the context of immunotherapy, CAFs induced by the IL-17/Act1/HIF1α pathway can initiate collagen deposition to enhance PD-L1 resistance, leading to a decrease in cytotoxic T cell infiltration [168]. Another CAF subtype, ecm-myCAF, has been found to upregulate PD-1 and CTLA4 protein levels in regulatory T lymphocytes (Tregs), increasing TGFβ-myCAF cellular content and mediating primary resistance to immunotherapy. Therefore, combining tumor-targeted therapy with CAF-targeted therapy has been considered a potential approach to address resistance. Examples of this approach, such as FAP5-DM1, an anti-FAP monoclonal antibody conjugated to maytansinoid, have shown long-lasting inhibition of tumor growth and complete regressions in xenograft models of multiple cancers [169]. Additionally, CAFs have been found to promote resistance to radiotherapy. Upon irradiation, CAFs are polarized towards the iCAF subtype via IL-1a with oxidative DNA damage, leading to p53-mediated therapy-induced senescence in iCAFs, which in turn results in chemoradiotherapy resistance and disease progression [170].

### tumor-restraining CAF

The tumor-promoting role of CAFs has been extensively studied; however, recent research suggests the existence of specific subtypes of CAFs with tumor-restraining characteristics termed cancer-restraining CAF (rCAF) [171]. The presence of rCAFs may pose a challenge to CAF-depleting therapies, as achieving a balance between tumor-promoting CAFs (pCAFs) and rCAFs is crucial. Despite this, only a few markers for identifying rCAFs have been identified. In 2019, Mizutani et al. discovered Meflin, a glycosylphosphatidylinositol-anchored protein and a marker of mesenchymal stromal/stem cells that maintain their undifferentiated state, to be expressed by PSCs, which are one of the sources of CAFs in PDAC. Meflin^+^ CAFs were found to be correlated with a favorable patient outcome, and Meflin deficiency promoted the alignment of stromal collagen fibers, which is considered an aggressive tumor signature [172]. Similarly, Bhattacharjee et al. showed that myCAF-expressed type I collagen suppresses tumor growth by mechanically restraining tumor spread, overriding signaling mechanisms induced by matrix stiffness [173]. And deletion of type I collagen accelerates the PDAC emergence via SOX9/Cxcl5 [174]. These findings suggest that collagen fibers produced by CAFs may contribute to their tumor-suppressing properties. In a transgenic mouse model, the depletion of α-SMA^+^ myofibroblasts in pancreatic cancer mechanistically resulted in a tumor with more progressive and invasive tumor feature. Paradoxically, this was accompanied by a decrease in overall immune infiltration and an increase in the frequency of FoxP3^+^ Treg cells [175]. These seemingly contradictory findings could be explained by the restraining influence of stromal components, particularly collagen deposition in models characterized by abundant collagen, such as PDAC [176]. Furthermore, the diverse subpopulations of CAFs, originating from different progenitors and influenced by distinct factors, exert varying effects on tumorigenesis depending on the specific context [172, 177]. Therefore, exploring the regulatory relationship between different contexts and CAF subtypes using multiple animal models may offer a promising avenue for breakthrough in therapies targeting CAFs.

## CAF-targeted cancer treatment

Cancer-targeted therapy has emerged as a viable and remarkable option for cancer patients since the early 2000s, encompassing a range of approaches such as drugs, viruses, and gene therapy [8, 9]. However, due to the genomic instability of cancer cells, the development of therapeutic resistance is inevitable. This has shifted the focus towards targeting non-tumor cells in the TME due to their relatively stable genetical nature, presenting a promising avenue for therapy [178]. Among these non-tumor cells, CAF have been recognized for their significant role in tumor progression and are now an emerging target for precise targeted therapy within the TME. Despite the potential of CAF-targeted therapy, there are ongoing challenges and obstacles. One major challenge it the lack of a specific CAF marker, which hinders the direct depletion of CAFs and makes it challenging to specifically target them. Additionally, there is a concern about potential adverse effect if normal tissue cells are unintentionally damaged during the therapy. Fortunately, as our understanding of CAF biology in cancer continues to advance, several preclinical studies and clinical trials have been reported, demonstrating promising results in this area. In the realm of CAF-targeted cancer therapy, NOX4 inhibition has emerged as a promising strategy, given its demonstrated efficacy in reversing the myCAF phenotype and facilitating intratumoral CD8^+^ T-cell infiltration in mouse models [179, 180]. Setanaxib (GKT137831), a pharmacologic NOX4 inhibitor, has successfully completed its phase I trial (NCT04327089) and is currently being investigated in combination with pembrolizumab in patients with recurrent or metastatic head and neck squamous cell carcinoma (NCT05323656). Moreover, LRRC15 has been identified as a particularly noteworthy myCAF biomarker due to its significant role in mediating CD8^+^ T cells infiltration and influencing immunotherapy response [63, 181]. A newly developed antibody–drug conjugate targeting LRRC15, known as ABBV-085, has demonstrated safety, tolerability, and promising anti-solid tumor activity in its phase I study (NCT02565758). These developments highlight the potential for more precise CAF-targeted therapy to be developed [182]. Further details on the evolving CAF-targeted therapies are shown in Table 2.

**Table 2:** List of CAF-targeted therapies with the associated mechanism, clinical or preclinical application in solid tumor, effect, and references

Therapy		Mechanism	e.g	Clinical pipeline in solid tumor	Effect	Ref
Reprogramming CAFs	TGF-β1 inhibition	Downregulate TGF-β/TGFβ-RI/Smad2	Galunisertib	Phase II	Suppress myCAF differentiation, but not iCAF; unlock innate and adaptive immune functions	[183–185]
		Block TGFβ1 signaling	Trihydroxyphenolics	Preclinical		[186]
		TGFβ-RII antibody; reduce IL-6/STAT3	2G8	Preclinical		[187]
	IL-1β inhibition	Block IL-1β	Canakinumab	Phase III	Reduce iCAF population separate from myCAF formation; decrease immune suppressive cells and increase infiltration of dendritic cells and effector T cells in TME	[188, 189]
			Gevokizumab	Phase I		
	Hedgehog inhibition	Inhibit SMO	LDE225	Phase II	Reduces myCAFs proliferation and CSC markers expression, while motivate iCAFs	[190][191, 192]
			Cyclopamine	Preclinical		
			Vismodegib	Phase II		
		Inhibit GLI	Curcumin	Phase III		[193–195]
			Resveratrol	Phase I		
			Epigallocatechin-3-gallate	Phase II		
			Arsenic trioxide	Phase II		
		Inhibit SHH	5E1	Preclinical		[196, 197]
			Genistein	Phase II		
	NOX4 inhibition	Abrogate TGF-β1-dependent ROS production	Setanaxib	Phase I	Revert myCAFs activation; promote tumor CD8^+^ T-cell infiltration	[179, 198]
	FGFR4 inhibition	Inhibit FGF19/FGFR4 Signaling	BLU9931	Preclinical	Inhibit EMT; reduce chemotherapy resistance of sorafenib	[199]
	AKT inhibition	Downregulate Bcl-2; inhibit AKT phosphorylation	Regorafenib	Clinical applied	Induce CAF apoptosis; affect macrophage infiltration	[200]
	Vitamins	Downregulate PI3K/AKT and Wnt/β-catenin	ATRA	Clinical applied	Suppress cell growth, G1/S transition, and colony formation	[201]
		Compete in vitamin D/vitamin D receptor/Smad3	Vitamin D	Clinical applied	Inhibit mesenchymal transition	[202]
CAF depletion		Inhibit FAP and dipeptidyl peptidase IV (DPPIV)	GluBoroPro dipeptide	Preclinical	Inhibit tumor cell proliferation; increase accumulation of collagen; decrease myCAF content; decrease blood vessel density in tumor	[203]
		Target CD10^+^GPR77^+^ CAFs	Anti-GPR77 antibody	Preclinical	Abolish tumor formation and restore tumor chemosensitivity	[60]
		kill cancer cells and non-neoplastic stromal populations via viral vaccines	ICOVIR15	Preclinical	Deplete CAF-like FAP^+^ pericytes as well as glioblastoma cells	[204]
CAF-directed drug conjugates		Anti-LRRC15 IgG1 antibody and the anti-mitotic drug monomethyl auristatin E	ABBV-085	Phase I	Produce M-phase cell cycle arrest relying on LRRC15^+^ CAFs	[205]
		Anti-FAP monoclonal antibody conjugated to maytansinoid	FAP5-DM1	Preclinical	Implicate mitotic arrest and apoptosis of malignant ECs; disruption of fibroblastic and vascular structures	[169]
Targeting CAF-associated immune evasion mechanisms	Wnt inhibition	Restore SOCS3/JAK2/STAT3 pathway	Anti-WNT2 mAb	Preclinical	Restore anti-tumor T cell responses; enhance anti-PD-1 efficacy by increasing active dendritic cells	[206]
	ATM inhibition	Target ataxia-telangiectasia mutated (ATM)	KU55933	Preclinical	Promoted intratumoral CD8 T-cell infiltration; potentiated the response to anti-PD-1 blockade and antitumor vaccination	[207]
	CXCL12/CXCR4 inhibition	Block CXCR4	AMD3100	Phase II	Impede CAF-mediated immunosuppression; release more T cells into the TME; increase tumor sensibility to anti-PD-L1 therapy	[73]
			Balixafortide	Phase II		
Targeting CAF-derived ECM		Inhibit fibroblast contraction and ECM deposition	Losartan	Phase II	Reduce liver metastases stiffening; increase the anti-angiogenic effects of bevacizumab	[208]

## Conclusion

The pivotal role of the TME in cancer progression has long been emphasized, with CAFs being the most well-described components. However, the heterogeneity of CAFs poses challenges for the application of clinical CAF-targeted therapy. This heterogeneity can be attributed to two main factors: (1) diverse origin of CAFs: CAFs can arise from different cell types, including quiescent fibroblasts, MSCs, adipocytes, and pericytes, through various activating pathways; (2) heterogeneous TME: The TME in various tumors is heterogeneous and can induce CAF activation via different signaling pathways, such as TGF-β, interleukin, PDGF, and CXCL12, among others. Phenotypically, this heterogeneity is reflected in the diverse expressing levels of multiple markers in distinct CAF clusters, as well as multifarious functions in ECM remodeling, inflammation, immunoregulation, and antigen presenting. The roles of CAFs in cancer progression, metastasis, and immunosuppression through both physical interactions and paracrine signaling have been extensively studied. Several therapeutic treatments targeting CAFs have been explored and put into clinical trials. However, none of these treatments have shown significant effectiveness or safety in clinical settings. Despite the challenges, the advanced discovery of specific markers and signaling pathways, such as LRRC15, offers hope that CAF-targeted therapy will progress from bench to bedside in the near future. As researchers continue to unravel the complexities of CAF heterogeneity and their precise roles in tumor biology, novel therapeutic strategies may emerge to effectively target CAFs and improve cancer treatment outcomes.

## Data Availability

Data sharing is not applicable to this article as no datasets were generated or analyzed in the study.
